# The National Pension Fund and Workers.—III

**Published:** 1890-12-06

**Authors:** 


### Passing Topics.

### THE NATIONAL PENSION FUND AND WORKERS.—III.

This is, perhaps, scarcely the place to enter very deeply into tables of pensions and premiums. Able papers have been prepared on behalf of the Fund, notably one by Mr. T. Ryan, the secretary of St. Mary's Hospital, which afford full information in the plainest possible form, and these, doubtless, together with the official book of figures, will be readily supplied upon application. Suffice to say here that the general idea is to make the pensions payable to retiring officials at 50, 55, or 60 years of age, and, if a stipulated addition to the premium be paid, to make provision besides for sick pay, which is to be continued, week by week, as long as necessary, or until the pension becomes payable.

It is also provided that if for any reason the policy-holder desires to make surrender, the whole of the payments in respect of the policy will be returned. Moreover, there is a proposal to allow interest at the rate of 2½ per cent. per annum, upon returned premiums, or at the same rate as the Post Office allows upon deposits, and if the Council should see their way to arrange for this crowning concession, no inducement would appear to be wanting to render this Fund the recognised friend and helpmate of hospital nurses. Indeed, we may easily believe that the day will come when not to be a policy holder will be considered a sure sign of improvidence, and of an inability to appraise the gravity of those contingencies of life which none can wholly guard against, but whose consequences may be sensibly mitigated.

Gathering up the observations we have offered in commendation of this institution, with a view to present our readers with one compact illustration, we cannot do better than endeavour to fill the spaces between the lines of the official book which lies before us, and to accentuate the moral its perusal is well calculated to convey. Let us put the case thus: A nurse entering upon her probationary period at the age of, say, 24 years, takes one year to determine that she has not mistaken her vocation. Assured upon that point she prudently desires to safeguard her future against the consequences of possible accidents and ailments, or if these be happily spared her, of that unavoidable feebleness of age which sooner or later must render her incapable of labour. To this end, she, in conjunction with the authorities of the hospital she is serving, makes application for a policy under which she will be granted a pension of £20 per annum upon attaining the age of 55 years, with a provision for sick pay when necessary in the interim. This policy, taken out when the applicant is 25 years of age will cost in premium twelve shillings and sixpence per month, of which the hospital pays one-half, leaving the nurse to pay six shillings and threepence.

In return for this sum, which no doubt represents at the outset a somewhat heavy charge upon her resources, but will become lighter in proportion as her position improves, she obtains on pension account the assurance of the yearly sum stated, plus her share of such profits as are earned by the society, and of the bonus fund, additions, calculated to enhance very considerably the value of the pension by the time she is qualified to receive it. It is estimated, roughly, that with all these advantages, a nurse will obtain a pension of £30 at a cost to herself of one of no more than £10, while similarly, governing bodies will secure pensions for their nurses by an outlay representing but a third of their actuarial value. If incapacitated by illness at any time before the pension is payable, she will be entitled to receive 15s. weekly by way of sick pay, and if she desires to surrender her policy she will receive back every penny she has paid plus, as we hope, the interest at 2½ per cent. Again, in event of premature death, the premiums paid and interest thereon are returnable to the nurse's legal representatives, or she may dispose of the sum by will, a provision made, we believe, by no other society. In this connection we may perhaps suggest to the council that it might prove an attractive feature to officials of the higher grades if, by payment of an additional premium, the one policy could be made to cover life assurance as well as to provide a pension. But even as it stands, the National Pension Fund offers a valuable service of which the more youthful of hospital workers and the governing bodies ought to be quick to take advantage.

Agricola.

[Donation Bonus Fund.—With reference to the statement in the article in The Hospital of the 22nd instant, we are requested to state that all matrons, sisters, and nurses who join the Fund will be eligible to participate in the Donation Bonus Fund.—Ed.]

